# Long-term occlusal tooth wear at the onset of permanent dentition

**DOI:** 10.1007/s00784-024-05550-4

**Published:** 2024-02-16

**Authors:** Thomas Schmid, Konstantinos Dritsas, Meret Gebistorf, Demetrios Halazonetis, Christos Katsaros, Nikolaos Gkantidis

**Affiliations:** 1 Department of Orthodontics and Dentofacial Orthopedics, School of Dental Medicine, University of Bern, Freiburgstrasse 7, CH-3010 Bern, Switzerland; 2 Department of Orthodontics, School of Dentistry, National and Kapodistrian University of Athens, 11527 Athens, Greece

**Keywords:** Tooth wear, Posterior teeth, Modern humans, Quantitative assessment, Long-term outcome

## Abstract

**Objectives:**

This study quantified the long-term occlusal wear in the natural posterior teeth and the associations per tooth type within the dentition.

**Methods:**

The sample included 70 orthodontically treated subjects (52 females and 18 males; median age, 14.3 years), followed for a 12.7-year period. They were consecutively selected with no tooth wear–related criteria. Post-treatment (T1) and follow-up dental casts (T2) were scanned and superimposed through three-dimensional methods. Occlusal wear volume of posterior teeth and tooth wear patterns were investigated through non-parametric statistics and analysis of covariance.

**Results:**

There were no significant differences between contralateral teeth. The average occlusal wear per posterior tooth was 2.3 mm^3^, with 65.2% of teeth showing values greater than 1 mm^3^. Males, mandibular teeth, and first molars exhibited slightly greater wear levels than females (median, 2.57 and 2.21 mm^3^, respectively; *p* = 0.005), maxillary teeth, and first or second premolars, respectively. In all first premolars and in the mandibular second premolars, the buccal cusps were primarily affected with no other distinct patterns. There were weak to moderate correlations between tooth types, apart from certain strong correlations detected in males.

**Conclusions:**

Posterior tooth wear was highly prevalent after a 13-year period starting at the onset of permanent dentition. The detected patterns are in accordance with the concept of canine guidance occlusion that is transforming into group synergy through function.

**Clinical relevance:**

The widespread tooth wear occurrence and the high intra- and inter-individual variability underline the need for individual patient monitoring to identify high-risk patients at early stages.

**Supplementary Information:**

The online version contains supplementary material available at 10.1007/s00784-024-05550-4.

## Introduction

Tooth wear is the natural process of continuing surface loss of enamel and, in severe cases, dentine or cementum. It begins with tooth emergence and progresses gradually over the years [1, 2]. Occlusal tooth wear refers specifically to the occlusal surfaces; therefore, occlusal contact and function are crucial in its development. Eventually, after a period of functioning, it affects the entire population to varying degrees [3]. While occlusal wear is physiological in the primary dentition, excessive tooth structure loss in the permanent dentition can cause aesthetic and functional impairment and is considered pathological [4, 5].

Men show higher occlusal wear severity and more affected teeth than women [2, 3, 6], possibly because they have stronger masseter muscles and produce higher bite forces [7, 8]. Certain malocclusion traits predispose to different progression rates or tooth wear patterns [9–12]. Reduction of tooth height through wear is more pronounced in the anterior than the posterior dentition [2, 13–15], but it is not clear if this corresponds to the total amount of tissue loss, as posterior teeth have wider occlusal surfaces than the anterior.

Aetiology of tooth wear is multifactorial. Functional factors include chewing patterns and habits, clenching, teeth grinding and bruxism, gastroesophageal reflux, bulimia, and aggressive brushing. Environmental factors include acidic foods, especially acidic drinks, energy drinks, acidic salad dressings, and the use of abrasive toothpastes or hard dental brushes [16]. The consumption of acidic drinks has increased steadily among young people in recent decades [17]. Regular consumption of acidic foods plays a significant role in the development of tooth wear [14, 15, 18]. Dental hard tissue structure characteristics also affect tooth wear progression [19–21].

Predisposing factors acting simultaneously can lead to severe tooth structure loss, requiring close monitoring and counselling, or complex management strategies [5, 22, 23]. This underscores the importance of diagnosing excessive tooth wear progression at an early stage to take preventive measures before more invasive interventions, such as crowns or extensive bite elevation with composite restorations, are required [15, 24, 25]. Preventive measures could include diet modification; removable splints, especially during sleep; cessation of harmful habits; or covering of tooth surfaces with composite resin to prevent further wear.

To date, the extent of the problem in the population, which will define the need for screening, monitoring, and intervention, as well as the aetiological factors contributing to it, have not been sufficiently investigated [3]. Most existing studies are descriptive and qualitative, subjective in nature, without accurate information on structure loss. This, along with the complex nature of the mechanisms that lead to tooth wear, confounds the outcomes. Long-term studies that accurately quantify the amount of tooth wear are scarce [3, 26–28], especially regarding the posterior dentition. Since life expectancy has increased significantly and one’s own teeth are to be preserved into old age, the need for further research is vital. The rising desire for aesthetically pleasing smiles and life-long function emphasises this need [29].

With recently developed 3D superimposition methods applied to intraoral digital models, tooth wear can be measured accurately and efficiently [3, 30–33]. In a previous study, we applied this methodology to quantify long-term tooth wear in the early phase of the anterior permanent dentition. Almost all individuals presented with tooth volume loss higher than 1 mm^3^ in at least one tooth, revealing the very broad occurrence in the population. In the present study, we aimed to quantify occlusal tooth wear over a 13-year period, focusing on the posterior dentition. The associations of tooth wear between different tooth types within the dentition were investigated as secondary outcomes.

## Materials and methods

The STROBE statement guidelines were followed for the reporting of this study [34].

### Ethical approval

This study was conducted according to the guidelines of the Declaration of Helsinki and approved by the Research Ethics Committee of the canton of Bern, Switzerland (Project-ID: 2019-00326). Prior to study commencement, a signed informed consent was obtained from all participants approving the use of their data.

### Sample

The present sample was selected from a pre-existing database that was originally created for a project with a different aim (gingival recession prevalence) [35]. The sample included orthodontically treated patients from a private orthodontic practice in Switzerland and was used in a previous publication by our group, testing anterior tooth wear development from adolescence to adulthood [3]. Thus, the primary sample characteristics are already reported in the previous publications [3, 35], but those that are necessary to allow deep comprehension of the present study by the readers will be repeated here.

All participants were assessed at two time points. Time point 1 (T1) was defined as the point when the participants have finished their orthodontic treatment. The outcome was stabilised with fixed retainers from canine to canine in both jaws, according to the clinic protocol. Time point 2 (T2) data were collected at a recall appointment performed at least 9 years later (between 2015 and 2016), consisting of alginate impressions and intraoral photos. The following inclusion criteria were used: (1) treated with fixed orthodontic appliances, (2) treated by the same orthodontist, (3) maxillary and mandibular retainers bonded immediately after active orthodontic treatment, and (4) non-syndromic patients. The exclusion criteria applied in the present study were as follows: (1) orthodontic retreatment, (2) T1 and T2 casts missing or of poor quality in the region of the posterior dental arch (e.g., artefacts like gypsum residues in the measurement area, due to air bubbles in the impression or small alginate recesses, chipped teeth on the plaster model, or accentuated occlusal or palato-lingual erosions), (3) periodontal disease other than gingival inflammation, and (4) teeth with occlusal, mesial, distal, or incisal fillings or with whole or partial crown reconstructions or heavily washed-out fissure sealants between T1 and T2. Moreover, a jaw was excluded from the analysis if less than two assessable crowns remained after the application of the exclusion criteria.

The T1 and T2 dental stone models generated through alginate impressions were scanned using a 3D laboratory surface scanner (stripe light/LED illumination; full dental arch accuracy < 20 µm; precision approximately 5 µm; Laboratory scanner D104a, Cendres + Métaux SA, Biel/Bienne, Switzerland). The subsequent maxillary and mandibular 3D surface models were exported as STL files (600,000–900,000 triangles each), which were processed to measure posterior tooth wear.

Visual inspection of intraoral photos and digital models was performed to assess eligibility. In case of doubt, the colour-coded distance maps of the teeth in question were reviewed after a best-fit superimposition, and the patients’ dental history was examined. The final decision was made after a discussion between two authors (TS, NG).

Out of the 72 participants of the previous study on anterior tooth wear [3], 70 were included in the present study (52 females; 18 males). Two patients had to be excluded, as well as four maxillary casts of an additional four patients. The reason was multiple composite restorations at the time of recall. Finally, tooth wear was measured on 677 individual teeth (Table 1). The median interval between the two time points T1 and T2 was 12.8 years (range 9–16 years). The median age of the participants at T1 was 14.3 years (range 12.0–23.2 years) (Table 2). All patients had acceptable occlusion at both time points, with the majority having a positive overjet and overbite not greater than 4 mm and an Angle class I occlusion. The detailed occlusal characteristics of the sample are provided in Supplementary Table S1.

**Table 1:** Teeth that comprised the posterior tooth wear measurement sample

First premolar		Second premolar		First molar	
Upper	Lower	Upper	Lower	Upper	Lower
*n* = 59 (46 F, 13 M)	*n* = 67 (49 F, 18 M)	*n* = 57 (45 F, 12 M)	*n* = 62 (46 F, 16 M)	*n* = 60 (47 F, 13 M)	*n* = 62 (48 F, 14 M)
56 R, 55 L	64 R, 65 L	55 R, 52 L	59 R, 55 L	49 R, 56 L	54 R, 57 L

*F* females, *M* males, *R* right, *L* left

**Table 2:** Demographic characteristics of the studied sample

	Mean age at T1 (range)	Mean follow-up duration (range)
Overall (*n* = 70)	14.8 y (12.0–23.2 y)	12.7 y (9.0–16.0 y)
Male (*n* = 18)	14.7 y (12.9–19.5 y)	12.7 y (10.6–14.5 y)
Female (*n* = 52)	14.8 y (12.0–23.2 y)	12.8 y (9.0–16.0 y)

*y* years

### Tooth wear assessment workflow

Tooth wear was measured using accurate superimposition techniques, developed by our team [30–32]. These were applied on corresponding single crowns of posterior teeth of the T1 and T2 models, using Viewbox 4 software (version 4.1.0.12 BETA, dHAL Software, Kifissia, Greece). The tooth crowns of six posterior mandibular and maxillary teeth (two premolars and the first molar of each quadrant) were selected on the T2 models. Each individual T2 tooth crown was set as a superimposition reference area for the calculation of tooth wear per tooth and was superimposed on the corresponding T1 crown, using the following settings: 20% estimated overlap of meshes, matching point to plane, exact nearest-neighbour search, 100% point sampling, exclude overhangs, and 50 iterations. A best-fit approximation of the corresponding T1 and T2 tooth crowns was accomplished using the software’s iterative closest point algorithm (ICP) [36], after an initial manual approximation that was performed to reduce the processing time.

The superimposed crowns of the T1 and T2 models were then sliced simultaneously using one or more planes, intersecting the models on non-occlusal, intact tooth crown structures, assisted by the visualisation of colour-coded distance maps. The resulting open shells of the occlusal parts underwent a standardised hole-filling process to create watertight models, enabling the calculation of their volume [31]. The T2 volume was then subtracted from the T1 volume, providing the amount of tooth wear in cubic millimetres. The measurement workflow and the evidence supporting it have been described thoroughly in previous publications [30–32] and are summarised in supplementary Fig. 1.

To enable valid comparisons between the calculated volumetric wear amounts at the different tooth types, the assessed occlusal surface area in each tooth type was measured in 39 randomly selected patients (38–41 teeth per tooth type).

The identification of potential specific tooth wear patterns per tooth type was performed through the visual inspection of colour-coded distance maps between superimposed tooth crowns by three investigators (T.S., K.D., and N.G.). Individual inspections were performed, and the final assessment was reached by consensus.

### Method error

The intra-rater reliability in tooth wear volumetric measurements was assessed through the repetition of the entire measurement process by the same examiner (T.S.). Six randomly selected patients were remeasured after a minimum period of 2 weeks. In total, 65 teeth were remeasured consisting of 23 first premolars, 22 second premolars, and 20 first molars.

For the intra-rater error on the assessment of the measured surface area per tooth type, 30 teeth (10 first premolars, 10 second premolars, and 10 first molars) of five randomly selected patients were remeasured by one investigator (T.S).

### Statistical analysis

Statistical analysis was performed in IBM SPSS statistics for Windows (Version 28.0. IBM Corp: Armonk, NY, USA). After testing data normality through the Kolmogorov–Smirnov and Shapiro–Wilk tests, as well as through the visualisation of data distribution graphs, significant deviations were detected in certain variables, and thus, non-parametric statistics were applied. The outcomes of interest were presented using exact measures and graphical representations.

Differences between the sides of the mouth (right or left) were tested separately for each tooth type and jaw using paired testing, and no significant differences were detected (Wilcoxon signed rank test, *p* > 0.05). Therefore, the average values of the right and left sides were calculated for each tooth type, and further analysis was performed per tooth type, without accounting for the right/left side factor.

Differences in the amount of tooth wear (dependent variable) among tooth types (fixed factor, 6 levels—upper first molar, upper second premolar, upper first premolar, lower first molar, lower second premolar, and lower first premolar) and between sexes (fixed factor, 2 levels—male and female) were tested using non-parametric analysis of covariance (Quade’s ANCOVA; general linear model, full factorial) [37]. The patient factor was set as a covariate to account for matching and clustering effects. Following significant results, pairwise comparisons were performed. Age at T1 and duration of the assessment period were not included in the model, based on exploratory tests through bivariate correlations with the different tooth types (Supplementary Table S2).

The association of tooth wear between the different tooth types was investigated through bivariate Spearman’s correlations. Possible correlations of the amount of tooth wear volume between anterior and posterior tooth types were investigated using the anterior wear data from a previous study of our group [3].

The amount of error was assessed through the absolute differences between repeated measurements. The testing of systematic errors and assessments of individual measurement errors were performed through the Bland–Altman method.

The level of significance was set at an alpha level of 0.05. A Bonferroni correction was applied when multiple pairwise comparisons were performed for similar outcomes. The unit of analysis was the tooth crown.

## Results

### Method error (intra-rater reliability)

The average intraoperator error was − 0.02 ± 0.14 mm^3^ for a total average wear value of 1.85 ± 1.84 mm^3^. Regarding each tooth type, the intraoperator error was 0.02 ± 0.15 mm^3^ for the first premolars, 0.00 ± 0.08 mm^3^ for the second premolars, and − 0.08 ± 0.14 mm^3^ for the first molars with average wear values of 1.07 ± 0.69 mm^3^, 1.25 ± 0.93 mm^3^, and 3.40 ± 2.45 mm^3^, respectively.

**Fig. 1:**
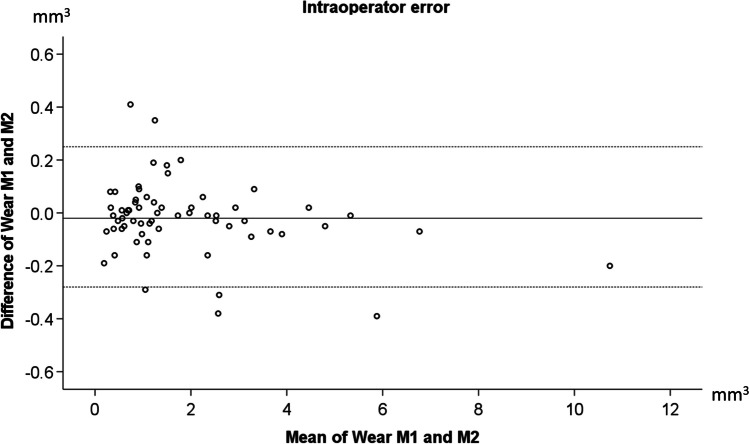
Bland–Altman plot of the differences between repeated occlusal wear measurements against their average. The continuous horizontal line represents the average of the differences (− 0.02 mm^3^), and the dashed horizontal lines represent the 95% limits of agreement (upper limit, 0.25; lower limit, − 0.28 mm^3^). M1, first measurement; M2, second measurement

The average of the absolute differences between repeated measurements was 0.09 ± 0.10 mm^3^, and the maximum difference detected was 0.41 mm^3^, with a maximum wear value of 10.74 mm^3^. Thus, the measurement error can be considered negligible. As evident in the Bland–Altman plot shown in Fig. 1, there were no systematic differences between repeated measurements (*n* = 65; paired *t*-test, *p* = 0.98; 95% limits of agreement: 0.25, − 0.28 mm^3^) and no evidence that the amount of error was related to the tooth wear amount.

The intra-rater error on the assessment of the measured surface area per tooth type was negligible. The average of the absolute differences between repeated measurements was 1.3 mm^2^ (SD 0.8) for an average surface area of 61.9 mm^2^ (SD 22.8).

### Tooth wear differences between contralateral sides

No significant differences between contralateral teeth of the same type were detected (Supplementary Table S3). Therefore, the amount of tooth wear of contralateral teeth of the same type was averaged for further analysis.

### Tooth wear amount (overall, between sexes, and among different tooth types)

**Fig. 2:**
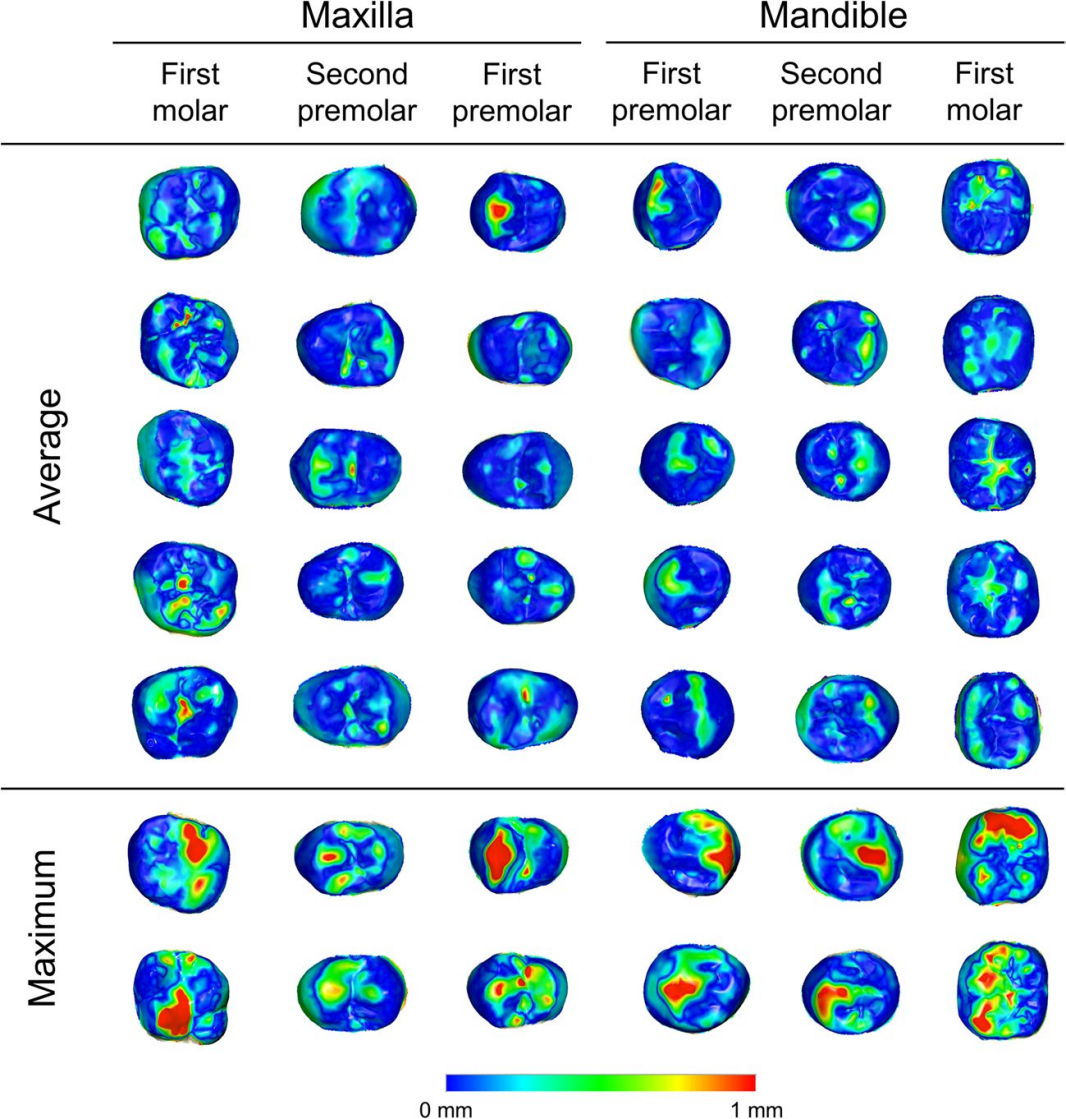
Colour-coded distance maps between best-fit approximated T1 and T2 tooth crowns representative of the average and maximum volumetric tooth wear amounts evident in the sample

The average occlusal wear per posterior tooth was 2.29 mm^3^. If we consider a loss of 1 mm^3^ in tooth volume as clinically relevant, 65.2% of all measured teeth presented a greater wear amount. Almost all participants (69 out of 70) had at least one posterior tooth with occlusal wear greater than 1 mm^3^. Representative teeth of the average and maximum wear amounts per tooth type are shown in Fig. 2.

**Fig. 3:**
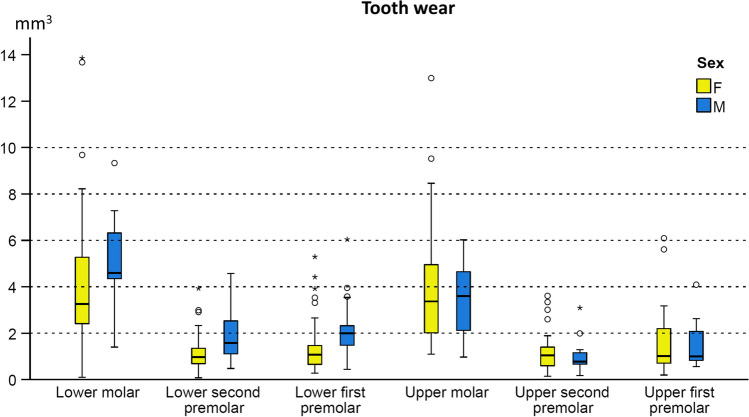
Box plots showing tooth wear for each tooth type and sex in the mandible and maxilla. The upper limit of the black line represents the maximum value, the lower limit is the minimum value, the box is the interquartile range (IQR), and the horizontal black line is the median value. Outliers are shown as black circles (further from the median more than 1.5 times the IQR) or asterisks in more extreme cases (further from the median more than 3 times the IQR)

ANCOVA testing revealed a significant effect of the tooth type (*p* < 0.001) and sex (*p* = 0.005) on the amount of occlusal tooth wear (Table 3 and Fig. 3). The effect of the tested factors on the tooth wear amount is shown in Supplementary Table S4. Males showed, on average, 2.57 mm^3^ (95% CI: 2.17–2.97 mm^3^) occlusal wear of the posterior teeth, whereas in females, the average tooth wear amounted to 2.21 mm^3^ (95% CI: 1.95–2.47 mm^3^). Overall, the pattern of wear severity per tooth type was similar across sexes (Fig. 3), with mandibular teeth showing consistently slightly higher wear amounts (Fig. 3, Supplementary Table S3). However, the sexual differences were primarily evident in the mandible, whereas no sexual dimorphism was detected for any maxillary tooth (Fig. 3, Supplementary Table S4).

**Table 3:** Results of the ANCOVA testing the effect of tooth type and sex on the detected tooth wear amount after controlling for the patient factor

Source	df	*F*	Sig
Corrected model	11	24.202	< 0.001
Intercept	1	1.721	0.190
Tooth type	5	34.817	< 0.001
Sex	1	8.133	0.005
Tooth type * sex	5	2.406	0.036

*R*-squared = 0.429 (adjusted *R*-squared = 0.411)

*df* degrees of freedom, *F F*-value, *Sig*. significance shown as *p*-values

The tooth that exhibited the highest amount of occlusal wear was the lower first molar (median, 3.40 mm^3^; IQR, 3.35 mm^3^), followed by the upper first molar (median, 3.10 mm^3^; IQR, 2.90 mm^3^). The premolars presented lower amounts of tooth wear, with the upper second premolar showing the lowest amount (median, 0.90 mm^3^; IQR, 1.00 mm^3^). The variability within tooth types was increasing with an increase in tooth wear amount (Fig. 3, Supplementary Table S3). In both jaws, the assessed occlusal surface area in the molars was approximately two times larger than that of the premolars (Supplementary Table S5).

Regarding the tooth wear amount within individuals, there were weak to moderate correlations between posterior tooth types, with no indication that the jaw factor had a mediating effect on these (average Spearman’s ρ, 0.36; range, 0.14–0.55; *p* < 0.05; Supplementary Table S6). Similar outcomes were evident when the two sexes were tested separately (Supplementary Table S7 and S8).

The tooth wear amount of posterior tooth types presented a weak to moderate correlation to that of the anterior teeth, when the entire sample was analysed (average Spearman’s ρ, 0.39; range, 0.28–0.49; *p* < 0.05; Supplementary Table S9). However, certain strong correlations were detected when the male patients were investigated separately (average Spearman’s ρ, 0.72; range, 0.54–0.80; *p* < 0.05; Supplementary Table S10). Relevant scatter plots of the strongest correlations are presented in Supplementary Figure S2. In the female subgroup, the analogous correlations were weak to moderate (average Spearman’s ρ, 0.35; range, 0.28–0.44; *p* < 0.05; Supplementary Table S11), and this was consistent in a subsample of 20 females with the highest tooth wear amounts, comparable to that of the males (Supplementary Table S12).

### Tooth wear patterns

Visual inspection of the colour-coded distance maps between corresponding T1 and T2 tooth crowns revealed some recurring tooth wear patterns on the posterior teeth, which were similar to contralateral teeth. The upper first premolars showed tooth wear more often and of higher degree on the buccal cusps, followed by the pits and fissures, the palatal cusps, and, finally, the mesial and distal marginal ridge areas, with no clear differences for the latter (Supplementary Figures S4 and S5). On the contrary, no specific pattern was more pronounced on the upper second premolars (Supplementary Figures S6 and S7), as well as on the upper first molars (Supplementary Figures S8 and S9), apart from the fact that the buccal cusps of the latter rarely showed increased wear amounts.

Regarding the lower teeth, the first premolars had similar patterns to the upper ones (Supplementary Figures S10 and S11). The mandibular second premolars presented tooth wear slightly more often and at a higher level on the buccal cusps, but this was not as pronounced as for the first premolars (Supplementary Figures S4, S5, S10-12, and S13). Finally, no specific tooth wear pattern was detectable at the lower first molars (Supplementary Figures S14 and S15).

## Discussion

Posterior tooth wear is an increasingly relevant phenomenon, as modern dentistry aims for life-long retention of natural teeth, enabling the long-term persistence of high oral health–related quality of life (OHRQoL) [38, 39]. Current evidence regarding long-term posterior tooth wear is insufficient. The present study quantified long-term occlusal wear, utilising previously validated, highly accurate superimposition techniques [3, 30–32]. Our findings revealed an average occlusal wear volume of 2.3 mm^3^ on the posterior permanent teeth, with 65% of them presenting values greater than 1 mm^3^, over a 13-year period extending from adolescence to adulthood. All but one participant had at least one such tooth, revealing the widespread occurrence in the population. There was high individual variation in tooth wear severity, which was mostly evident in molars that showed the highest amount of tooth wear.

The visualisation of individual three-dimensional colour-coded distance maps per tooth type revealed few recurring wear patterns that were symmetrical between contralateral teeth. These primarily concerned the buccal cusps of the upper and lower first premolars and might be related to the progression of the canine guidance occlusion achieved through orthodontic treatment to group function [40]. The previously observed increased canine wear corroborates this argument [3]. A similar tendency, but of lower intensity, was evident on the mandibular second premolars, and no clear pattern was detectable on the molars, despite the higher wear amount. This concept seems reasonable when considering the longer buccal premolar cusps, compared to the palatal, and the short molar cusps of equal height. To date, there are no similar assessments in the literature, but there is indirect evidence indicating increased intra- and inter-individual variation in tooth wear phenotypes [26, 28], as shown here.

To our knowledge, this is the first quantitative study thoroughly investigating long-term posterior tooth wear. A study by O’Toole et al. investigated tooth wear in the dentition after a 3-year interval [26]. The average occlusal wear of the first molars was 2.4 mm^3^, whereas our study detected an average of 4.0 mm^3^. Considering the four times longer assessment period of our study, it seems that slower wear progression was evident. This difference can be attributed to clinical, cultural, and lifestyle differences in the tested populations or to variations in measurement methods. Most importantly, our population did not have specific tooth wear treatment needs whereas that of O’Toole et al. [26] was referred for tooth wear management [41]. Overall, there is very limited evidence on tooth wear quantification. Several studies use qualitative indices, which may be reliable for large lesions, but have questionable performance when lesions are limited to enamel [42]. Moreover, few qualitative scoring systems have been adequately tested in terms of reliability and ease of application [43]. Thus, there is a need for accurate quantitative outcomes to better understand tooth wear development and enable timely detection, prior to the establishment of associated adverse effects.

The amount of posterior tooth wear was slightly higher in males than in females, with differences limited to the mandible. This contradicts previous findings of clearly higher wear in males on all anterior teeth [3]. Moreover, occlusal wear between posterior tooth types was weakly correlated, in contrast to stronger correlations between anterior teeth. These findings suggest that at the onset of permanent dentition, wear-related risk factors may have a lesser effect on the posterior than on the anterior teeth, and early tooth wear detection should be focused on the anterior teeth. A possible explanation might be the posterior disocclusion during mastication or parafunctional activities, such as bruxism, due to the canine guidance or group synergy effects. As a result, the front teeth seem to be the first uniformly affected by tooth wear, especially in males, who also exhibit higher bruxism prevalence [44]. Other erosion-related factors also affect more of the males, the anterior dentition, and the molars [45]. Higher tooth wear levels or follow-up times may be needed to investigate possible sexual differences in posterior tooth wear.

When posterior tooth wear was correlated with anterior wear, strong correlations were detected only in males. This supports the notion that sex is a risk factor for tooth wear development [2, 6, 45]. It might also indicate that at the early phases of wear development, correlations between different tooth types might be weak for the reasons discussed above, but as the phenomenon progresses, it develops more uniformly throughout the dentition. Due to anatomical considerations related to functional or parafunctional occlusal contacts, the anterior dentition might be initially more intensively and uniformly affected, but after a certain progression point, the entire dentition is involved. Further progress might lead to the flattening of all incisal/occlusal surfaces throughout the dentition. This notion needs to be confirmed in longitudinal samples following wear development from early to progressed stages.

To further elaborate on this, we correlated the posterior with the anterior tooth wear in the 20 females with the highest wear amount, which was comparable to that of males. In contrast to males, no positive correlations were detected, and the only significant correlation was negative (Supplementary Table S12, Supplementary Figure S3). This suggests a different wear progression pattern between sexes, perhaps related to the underlying etiological factors. For example, in males, factors with generalised effects, such as bruxism, might be predominant, as opposed to more local factors in females, such as parafunctional habits. This needs further investigation in large prospective cohorts investigating the progression of minimal to severe wear.

Tooth wear does not always impact OHRQoL, and this does not depend on wear severity. However, associated risk factors (either precursors or consequences), such as tooth sensitivity, tooth appearance, or a diet with high amounts of acidic food can severely impact OHRQoL [46]. This showcases the complexity and multifactorial nature of this condition and the need to establish proper diagnostic tools for its early detection at an individual level. This would be difficult without accurate 3D quantification and visualisation tools, which aid precise diagnosis but also patient-doctor communication [3, 31]. Patients can see the actual condition, extent, and location of the problem, make informed treatment decisions, and follow the tooth or material wear progression, increasing their awareness and commitment to treatment. It should be noted that at the early stages of tooth wear development, sharp incisal edges or tooth cusps are smoothened—potentially relevant for aesthetics, masticatory efficiency, occlusal guidance, orthodontic correction stability, etc.—whereas later on, tooth wear affects relatively flattened surfaces, with possibly less noticeable outcomes. Wear management strategies range from monitoring/counselling at predefined time periods for the modification of contributing factors, such as diet, to preventive non-invasive measures, such as occlusal splints, and more invasive restorative strategies. The high intra- and inter-individual variability in wear progression showcases the difficulty in identifying high-risk teeth and patients at an early stage and also in determining the most appropriate treatment approach. Material wear also needs monitoring to test the integrity of restorations and their effects on natural antagonist teeth [47].

Molars showed approximately four times higher volumetric wear than premolars. However, when assessing the individual colour-coded distance maps, vertical differences were not that extensive. This could be due to the higher morphological complexity of the molar surfaces, along with the two times larger analysed occlusal molar surface.

The present study provided detailed 3D information on the long-term posterior tooth wear development in modern humans, from the early stages till the establishment of permanent dentition. The sample can be considered representative of the general population [48], since it comprised a consecutive selection of successfully treated, regular orthodontic patients [3] that fulfilled certain, wide eligibility criteria, not related to tooth wear. The endemic malocclusion prevalence along with the broad access of the Swiss population to orthodontic treatment supports this argument [49]. Other limitations related to the restricted geographical representation, the retrospective data collection at the T1 phase, and the lack of information on tooth wear–related factors have been thoroughly discussed in a previous publication [3]. The results of this study and their implications may be only cautiously extended to non-orthodontically treated patients and older individuals, as well as residents of other geographic locations.

## Conclusions

The incidence of tooth wear that might require monitoring and intervention was high already in early adulthood. There was variability between sexes, tooth types, and within the dentition, which was increasing with an increase in tooth wear amount. At the onset of permanent dentition, the few detected patterns were in accordance with the concept of a canine guidance occlusion that is transforming into group synergy through function.

The present outcomes underline the need for individual patient monitoring at early stages. Future research should elaborate further on tooth wear development in the presence of certain contributing factors and assess sexual differences in longer assessment periods.

### Supplementary Information

Below is the link to the electronic supplementary material.

Supplementary file1 (PDF 2544 KB)

## Data Availability

All data are available in the main text or the extended data. The protocols and datasets generated and/or analysed during the current study are available from the corresponding author on reasonable request.
