# Supplementary material for: Long-term occlusal tooth wear at the onset of permanent dentition
Source: Clin Oral Investig. 2024 Feb 16;28(2):155. doi: 10.1007/s00784-024-05550-4 (PMC10873235; doi:10.1007/s00784-024-05550-4)
Supplement: Supplementary file 1 — Supplementary file1 (PDF 2544 KB) [file 784_2024_5550_MOESM1_ESM.pdf]

Clinical Oral Investigations - Supplementary material

Long term occlusal tooth wear at the onset of permanent dentition.

Thomas Schmid[1], Konstantinos Dritsas[1], Meret Gebistorf[1], Demetrios Halazonetis[2], Christos Katsaros[1], Nikolaos Gkantidis[1*]

[1]*Department of Orthodontics and Dentofacial Orthopedics, School of Dental Medicine, University of Bern, CH-3010 Bern, Switzerland*

[2]*Department of Orthodontics, School of Dentistry, National and Kapodistrian University of Athens, GR-11527 Athens, Greece*

*Corresponding author: nikolaos.gkantidis@unibe.ch

Supplementary Table S1. Occlusal characteristics of the studied sample.

		**T1**	**T2**
Angle Class[1]	Class I	68	62
	Class II	1 (unilateral)	8 (7 unilateral, 1 bilateral)
	Class III	1 (bilateral)	1 (bilateral)
Overjet[2]	Normal	70	68
	Increased	0	2
Overbite[3]	Normal	63	57
	Reduced *	7	13

[1]Class I: Molar relationship less than ¼ cusp deviation from Class I (in cases of asymmetrical extractions, premolar and canine occlusion was also considered).
[2]Normal: 1–4 mm, Increased: >4 mm
[3]Normal: 1–4 mm, Reduced: <1 mm.
*Only one patient at T2 had a negative overbite and a bilateral class III. In all other cases, the overbite was between 0 and 1 mm.
T1: time point at end of orthodontic treatment. T2: recall time point was on average 12.7 years after T1.

Supplementary Table S2. Spearman's correlation of the initial patient's age (T1) and the duration of the assessment period with the tooth wear amount detected for the different posterior tooth types.

		Upper first molar	Upper second premolar	Upper first premolar	Lower first molar	Lower second premolar	Lower first premolar
Age at T1	Spearman's ρ	-0.06	-0.13	-0.01	-0.23	-0.18	0.14
	P-value	0.632	0.349	0.982	0.076	0.156	0.256
	N	60	57	59	62	62	67
Assessment period	Spearman's ρ	0.22	0.06	0.16	-0.01	0.06	0.18
	P-value	0.088	0.646	0.219	0.976	0.633	0.133
	N	60	57	59	62	62	67

*Significant correlations with $p < 0.05$

Supplementary Table S3. Differences in tooth wear (mm^3) measured for the different posterior tooth types at the right and left sides of the mouth and overall.

		N	Median	IQR	P-value*		N	Median	IQR
Upper first molar	right	49	3.14	3.37	0.192	Overall	105	3.10	2.90
	left	56	2.97	2.73					
Upper second premolar	right	55	0.80	1.07	0.240	Overall	107	0.90	1.00
	left	52	0.96	1.12					
Upper first premolar	right	56	0.98	1.20	0.277	Overall	111	1.00	1.25
	left	55	0.97	1.77					
Lower first molar	right	54	3.68	3.08	0.352	Overall	111	3.40	3.35
	left	57	3.18	3.86					
Lower second premolar	right	59	1.10	1.51	0.963	Overall	114	1.10	1.10
	left	55	1.12	0.90					
Lower first premolar	right	64	1.38	1.42	0.609	Overall	131	1.20	1.40
	left	65	1.12	1.65					

IQR: Interquartile range

*Wilcoxon signed rank test, P = 0.01, Bonferroni correction applied

Supplementary Table S4. Parameter estimates indicating the effect of tooth type and sex on tooth wear amount (dependent variable).

Parameter	B	95% Confidence Interval		Sig.
		Lower Bound	Upper Bound	
Intercept	-80.58	-126.81	-34.35	<0.001
Lower molar (ref.: upper second premolar)	212.33	149.34	275.33	<0.001
Lower first premolar (ref.: upper second premolar)	105.92	46.24	165.60	<0.001
Lower second premolar (ref.: upper second premolar)	78.81	17.66	139.97	0.012
Upper molar (ref.: upper second premolar)	169.87	105.77	233.98	<0.001
Upper first premolar (ref.: upper second premolar)	42.24	-20.75	105.24	0.188
Female (ref.: male)	15.72	-36.31	67.75	0.553
Lower molar * Female (ref.: lower molar * male)	-62.59	-133.82	8.63	0.085
Lower first premolar * Female (ref.: lower first premolar * male)	-93.99	-162.22	-25.76	0.007
Lower second premolar * Female (ref.: lower second premolar * male)	-74.68	-144.44	-4.91	0.036
Upper molar * Female (ref.: upper molar * male)	-14.77	-87.05	57.52	0.688
Upper first premolar * Female (ref.: upper first premolar * male)	-20.48	-91.95	50.99	0.573

ref.: reference

Supplementary Table S5. Measured occlusal surface area per tooth type in mm^2.

	Mean	SD
Upper first molar	92.60	8.13
Upper second premolar	49.40	5.72
Upper first premolar	48.36	4.45
Lower first molar	92.75	9.43
Lower second premolar	46.38	4.13
Lower first premolar	41.53	3.07

Supplementary Table S6. Spearman's correlations of tooth wear amount between the different posterior tooth types in the entire sample.

		Upper first molar	Upper second premolar	Upper first premolar	Lower first molar	Lower second premolar	Lower first premolar
Upper first molar	Spearman's ρ		0.28	0.34	0.20	0.14	0.35
	P-value		0.041*	0.012*	0.142	0.295	0.007*
	N		53	53	56	54	57
Upper second premolar	Spearman's ρ	0.28		0.55	0.39	0.47	0.40
	P-value	0.041*		<0.001*	0.003*	<0.001*	0.002*
	N	53		50	54	54	55
Upper first premolar	Spearman's ρ	0.34	0.55		0.22	0.25	0.53
	P-value	0.012*	<0.001*		0.096	0.070	<0.001*
	N	53	50		56	55	58
Lower first molar	Spearman's ρ	0.20	0.39	0.22		0.48	0.35
	P-value	0.142	0.003*	0.096		<0.001*	0.006*
	N	56	54	56		57	59
Lower second premolar	Spearman's ρ	0.14	0.47	0.25	0.48		0.46
	P-value	0.295	<0.001*	0.070	<0.001*		<0.001*
	N	54	54	55	57		59
Lower first premolar	Spearman's ρ	0.35	0.40	0.53	0.35	0.46	
	P-value	0.007*	0.002*	<0.001*	0.006*	<0.001*	
	N	57	55	58	59	59	

*Significant correlations with $p < 0.05$

Supplementary Table S7. Spearman's correlations of tooth wear amount between the different posterior tooth types in males.

		Upper first molar	Upper second premolar	Upper first premolar	Lower first molar	Lower second premolar	Lower first premolar
Upper first molar	Spearman's ρ		-0.31	0.32	0.10	0.28	0.29
	P-value		0.355	0.340	0.770	0.377	0.334
	N		11	11	11	12	13
Upper second premolar	Spearman's ρ	-0.309		0.13	0.46	0.55	0.18
	P-value	0.355		0.725	0.154	0.063	0.579
	N	11		10	11	12	12
Upper first premolar	Spearman's ρ	0.32	0.13		0.41	0.49	0.58
	P-value	0.340	0.725		0.190	0.089	0.030*
	N	11	10		12	13	14
Lower first molar	Spearman's ρ	0.10	0.46	0.41		0.71	0.33
	P-value	0.770	0.154	0.190		0.004*	0.249

	N	11	11	12		14	14
Lower second premolar	Spearman's ρ	0.28	0.55	0.49	0.71		0.50
	P-value	0.377	0.063	0.089	0.004*		0.047*
	N	12	12	13	14		16
Lower first premolar	Spearman's ρ	0.29	0.18	0.58	0.33	0.50	
	P-value	0.334	0.579	0.030*	0.249	0.047*	
	N	13	12	14	14	16	

*Significant correlations with $p < 0.05$

Supplementary Table S8. Spearman's correlations of tooth wear amount between the different posterior tooth types in females.

		Upper first molar	Upper second premolar	Upper first premolar	Lower first molar	Lower second premolar	Lower first premolar
Upper first molar	Spearman's ρ		0.40	0.33	0.26	0.13	0.31
	P-value		0.009*	0.033*	0.083	0.397	0.038*
	N		42	42	45	42	44
Upper second premolar	Spearman's ρ	0.40		0.62	0.40	0.50	0.52
	P-value	0.009*		0.000*	0.008*	0.001*	0.000*
	N	42		40	43	42	43
Upper first premolar	Spearman's ρ	0.33	0.62		0.17	0.18	0.53
	P-value	0.033*	0.000*		0.277	0.249	0.000*
	N	42	40		44	42	44
Lower first molar	Spearman's ρ	0.26	0.40	0.17		0.26	0.25
	P-value	0.083	0.008*	0.277		0.093	0.103
	N	45	43	44		43	45
Lower second premolar	Spearman's ρ	0.13	0.50	0.18	0.26		0.25
	P-value	0.397	0.001*	0.249	0.093		0.109
	N	42	42	42	43		43
Lower first premolar	Spearman's ρ	0.31	0.52	0.53	0.25	0.25	
	P-value	0.038*	0.000*	0.000*	0.103	0.109	
	N	44	43	44	45	43	

*Significant correlations with $p < 0.05$

Supplementary Table S9. Spearman's correlations of tooth wear amount between anterior versus posterior teeth in the entire sample.

		Upper first molar	Upper second premolar	Upper first premolar	Lower first molar	Lower second premolar	Lower first premolar
Upper canine	Spearman's ρ	0.083	0.188	0.385	0.366	0.295	0.494
	P-value	0.530	0.160	0.003*	0.004*	0.020*	0.000*
	N	59	57	58	61	62	66
Upper lateral incisor	Spearman's ρ	-0.148	0.071	0.202	0.281	0.171	0.392
	P-value	0.273	0.606	0.132	0.031*	0.192	0.001*
	N	57	55	57	59	60	63
Upper central incisor	Spearman's ρ	0.171	0.293	0.337	0.456	0.209	0.434
	P-value	0.208	0.031*	0.013*	0.000*	0.119	0.001*
	N	56	54	54	57	57	60
Lower canine	Spearman's ρ	0.076	0.217	0.358	0.288	0.290	0.430
	P-value	0.586	0.129	0.009*	0.033*	0.032*	0.001*
	N	54	50	52	55	55	60
Lower lateral incisor	Spearman's ρ	0.189	0.280	0.284	0.312	0.215	0.347
	P-value	0.180	0.054	0.043*	0.023*	0.119	0.008*
	N	52	48	51	53	54	57
Lower central incisor	Spearman's ρ	0.209	0.385	0.223	0.490	0.455	0.467
	P-value	0.154	0.009*	0.140	0.000*	0.001*	0.000*
	N	48	45	45	48	49	52

*Significant correlations with $p < 0.05$

Supplementary Table S10. Spearman's correlations of tooth wear amounts between anterior versus posterior teeth in male subjects.

		Upper first molar	Upper second premolar	Upper first premolar	Lower first molar	Lower second premolar	Lower first premolar
Upper canine	Spearman's ρ	0.225	0.137	0.789	0.121	0.050	0.445
	P-value	0.459	0.672	0.001*	0.680	0.854	0.064
	N	13	12	14	14	16	18
Upper lateral incisor	Spearman's ρ	0.007	0.405	0.363	0.102	0.095	0.312
	P-value	0.983	0.216	0.223	0.741	0.737	0.240
	N	12	11	13	13	15	16
Upper central incisor	Spearman's ρ	-0.282	0.802	0.382	0.327	0.189	0.464
	P-value	0.401	0.005*	0.247	0.326	0.555	0.095
	N	11	10	11	11	12	14
Lower canine	Spearman's ρ	0.030	0.418	0.445	-0.355	0.017	0.125
	P-value	0.934	0.262	0.170	0.284	0.957	0.657

	N	10	9	11	11	13	15
Lower lateral incisor	Spearman's ρ	-0.617	0.719	0.042	-0.122	-0.168	-0.095
	P-value	0.077	0.045*	0.907	0.738	0.601	0.748
	N	9	8	10	10	12	14
Lower central incisor	Spearman's ρ	0.176	0.778	0.264	0.515	0.485	0.543
	P-value	0.627	0.014*	0.433	0.105	0.093	0.037*
	N	10	9	11	11	13	15

*Significant correlations with $p < 0.05$

Supplementary Table S11. Spearman's correlations of tooth wear amount between anterior versus posterior teeth in female subjects.

		Upper first molar	Upper second premolar	Upper first premolar	Lower first molar	Lower second premolar	Lower first premolar
Upper canine	Spearman's ρ	0.056	0.352	0.348	0.273	0.230	0.373
	P-value	0.712	0.018*	0.021*	0.063	0.125	0.009*
	N	46	45	44	47	46	48
Upper lateral incisor	Spearman's ρ	-0.166	0.200	0.185	0.247	0.067	0.302
	P-value	0.275	0.192	0.229	0.098	0.660	0.039*
	N	45	44	44	46	45	47
Upper central incisor	Spearman's ρ	0.276	0.359	0.305	0.401	0.147	0.437
	P-value	0.067	0.017*	0.047*	0.006*	0.334	0.002*
	N	45	44	43	46	45	46
Lower canine	Spearman's ρ	0.082	0.350	0.364	0.242	0.220	0.342
	P-value	0.595	0.025*	0.019*	0.113	0.161	0.021*
	N	44	41	41	44	42	45
Lower lateral incisor	Spearman's ρ	0.331	0.314	0.304	0.308	0.113	0.364
	P-value	0.030*	0.049*	0.053	0.045*	0.478	0.016*
	N	43	40	41	43	42	43
Lower central incisor	Spearman's ρ	0.204	0.428	0.178	0.336	0.284	0.326
	P-value	0.220	0.009*	0.314	0.042*	0.093	0.049*
	N	38	36	34	37	36	37

*Significant correlations with $p < 0.05$

Supplementary Table S12. Spearman's correlations of tooth wear amount between anterior versus posterior teeth in the 20 female subjects with the highest average occlusal wear per tooth (2.86±1.06 mm^3). The wear amount of this subgroups was comparable to that of male subjects (2.71±0.91 mm^3; unpaired t-test, P = 0.642).

		Upper first molar	Upper second premolar	Upper first premolar	Lower first molar	Lower second premolar	Lower first premolar
Upper canine	Spearman's ρ	-0.309	0.114	-0.097	0.200	0.312	0.071
	P-value	0.244	0.685	0.721	0.458	0.240	0.779
	N	16	15	16	16	16	18
Upper lateral incisor	Spearman's ρ	-0.324	0.114	0.029	-0.009	0.176	0.184
	P-value	0.222	0.685	0.914	0.974	0.513	0.480
	N	16	15	16	16	16	17
Upper central incisor	Spearman's ρ	0.174	0.048	-0.121	0.143	-0.066	0.069
	P-value	0.520	0.864	0.656	0.598	0.807	0.793
	N	16	15	16	16	16	17
Lower canine	Spearman's ρ	-0.644	0.209	-0.321	0.121	0.424	-0.086
	P-value	0.007*	0.494	0.243	0.666	0.131	0.743
	N	16	13	15	15	14	17
Lower lateral incisor	Spearman's ρ	-0.049	0.104	-0.150	0.084	0.253	-0.084
	P-value	0.858	0.734	0.594	0.766	0.383	0.757
	N	16	13	15	15	14	16
Lower central incisor	Spearman's ρ	-0.352	0.084	-0.196	0.343	0.552	0.132
	P-value	0.239	0.795	0.542	0.276	0.063	0.668
	N	13	12	12	12	12	13

*Significant correlations with $p < 0.05$

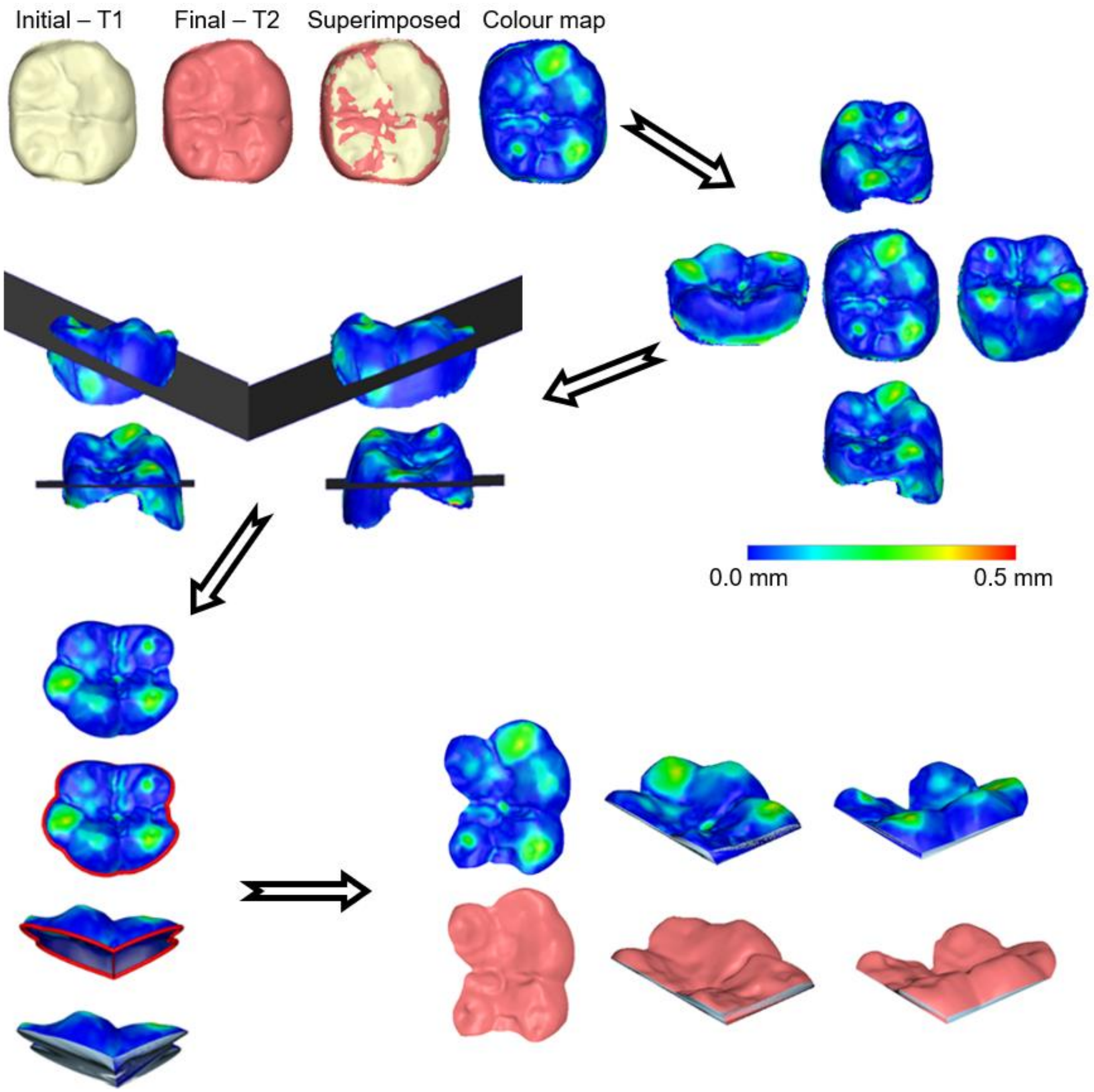

Supplementary Figure S1. Occlusal tooth wear measurement workflow: 1. Positioning of initial model (T1, yellow) adjacent to the final model (T2, red) to check for occlusal artefacts through visual inspection under high magnification. 2. Manual approximation of the T1 and T2 crowns to facilitate the subsequent automatic best-fit superimposition. 3. Application of an iterative closest point (ICP) algorithm for the best-fit approximation of the corresponding T1 and T2 crowns based on pre-defined settings. 3. Creation of color-coded distance maps on the T1 crowns to accurately visualize and quantify their differences from the corresponding, superimposed T2 crowns. A second detection session of potential artifacts was performed at this stage. 4. Eligible models were further processed by cutting the superimposed crowns simultaneously, using various cutting planes, aiming to isolate comparable occlusal crown parts of interest that differ only in the worn occlusal part. 5. Application of a standardized hole-filling process on the resulting open shells of the T1 and T2 occlusal parts, to create comparable watertight models, whose volumes would differ only due to the worn occlusal parts. 6. Subtraction of the T2 volume from the T1 volume provided the amount of occlusal tooth wear in the assessed T1 to T2 time period (12.7 years on average in the tested sample) and visualization of the color-coded distance maps on the T1 crowns, depicting their differences from the corresponding T2 crowns, provided the precise quantification of the spatial distribution of tooth wear per case in high detail.

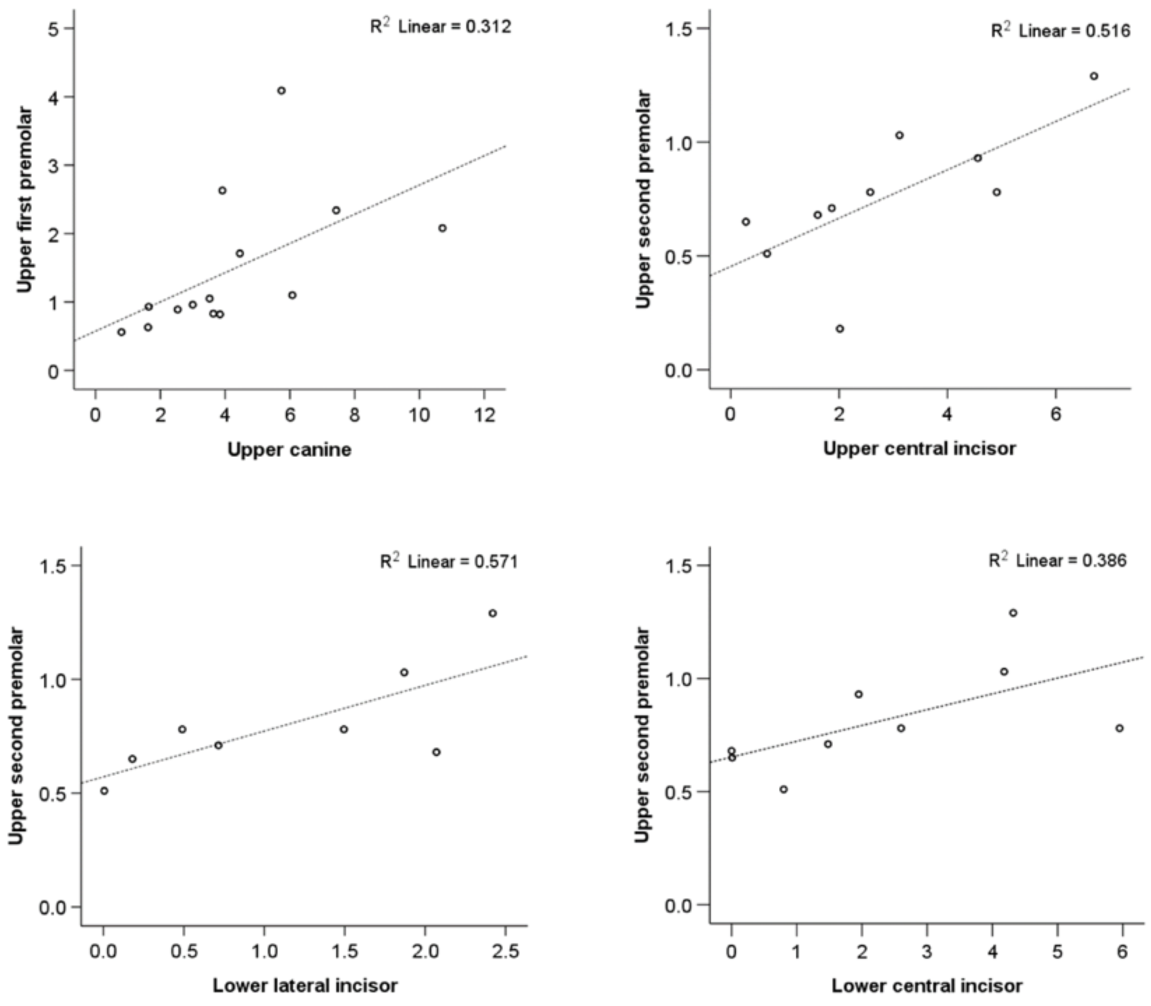

Supplementary Figure S2. Scatter plots of the strongest Spearman's correlations of tooth wear amount between the different tooth types in anterior versus posterior teeth in male subjects.

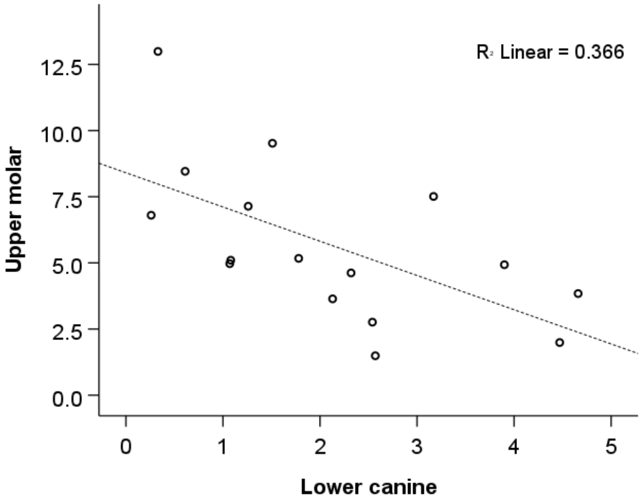

Supplementary Figure S3. Scatter plot of the strongest Spearman's correlation of tooth wear amount between the different tooth types in anterior versus posterior teeth in the 20 female subjects with the highest average occlusal wear per tooth.

Right maxillary first premolar

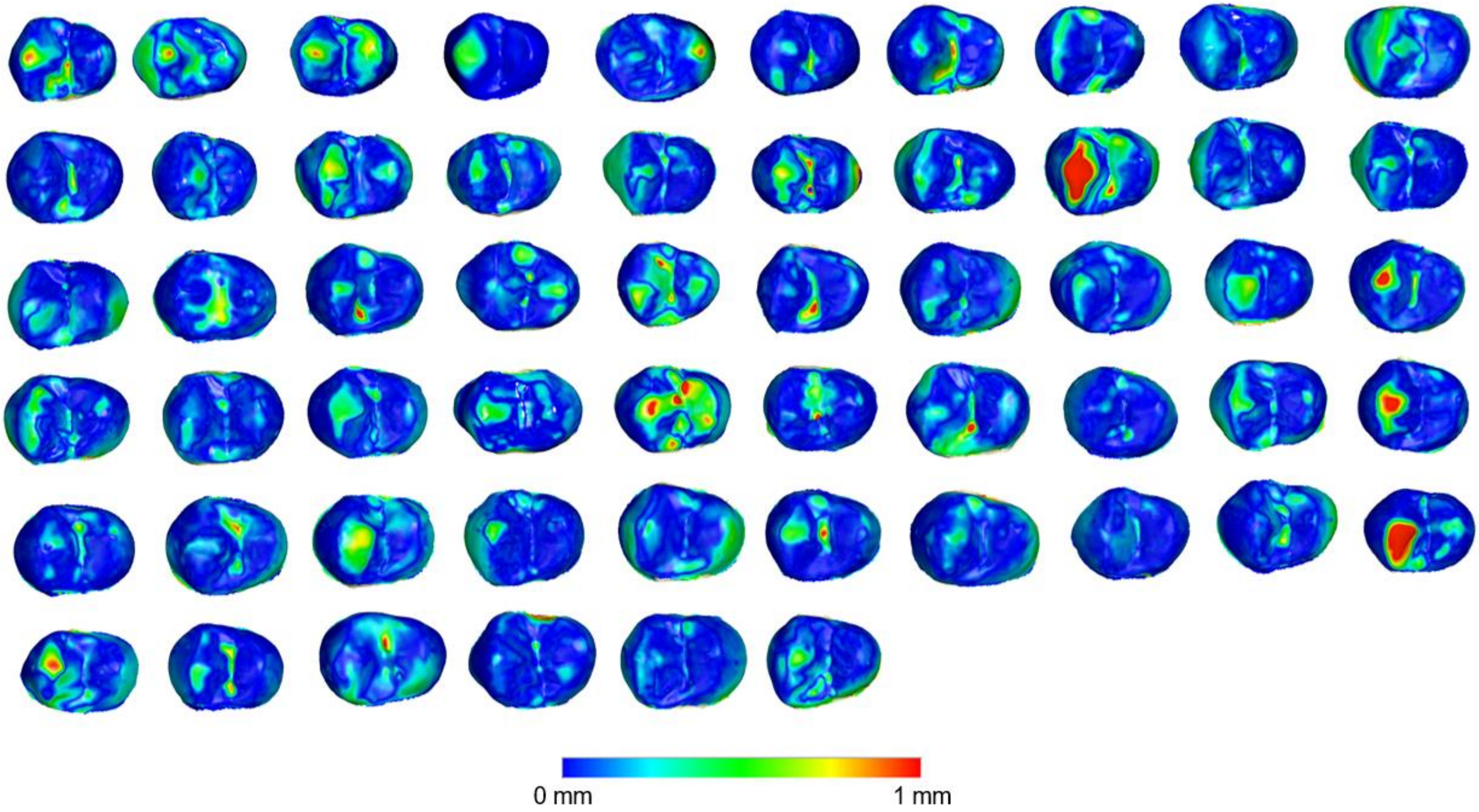

Supplementary Figure S4. Colour-coded distance maps illustrating all tooth wear patterns observed at the right maxillary first premolars.

Left maxillary first premolar

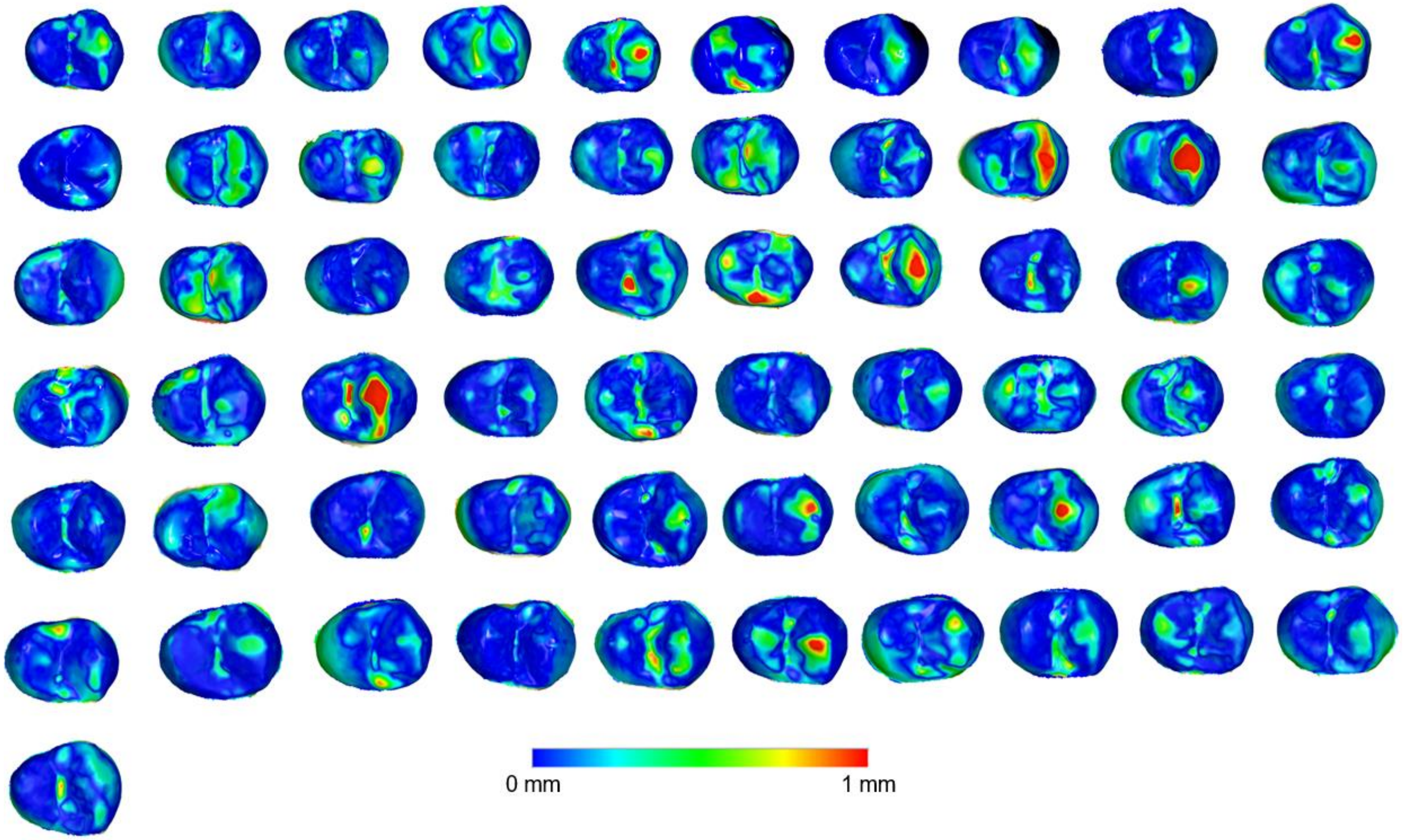

Supplementary Figure S5. Colour-coded distance maps illustrating all tooth wear patterns observed at the left maxillary first premolars.

Right maxillary second premolar

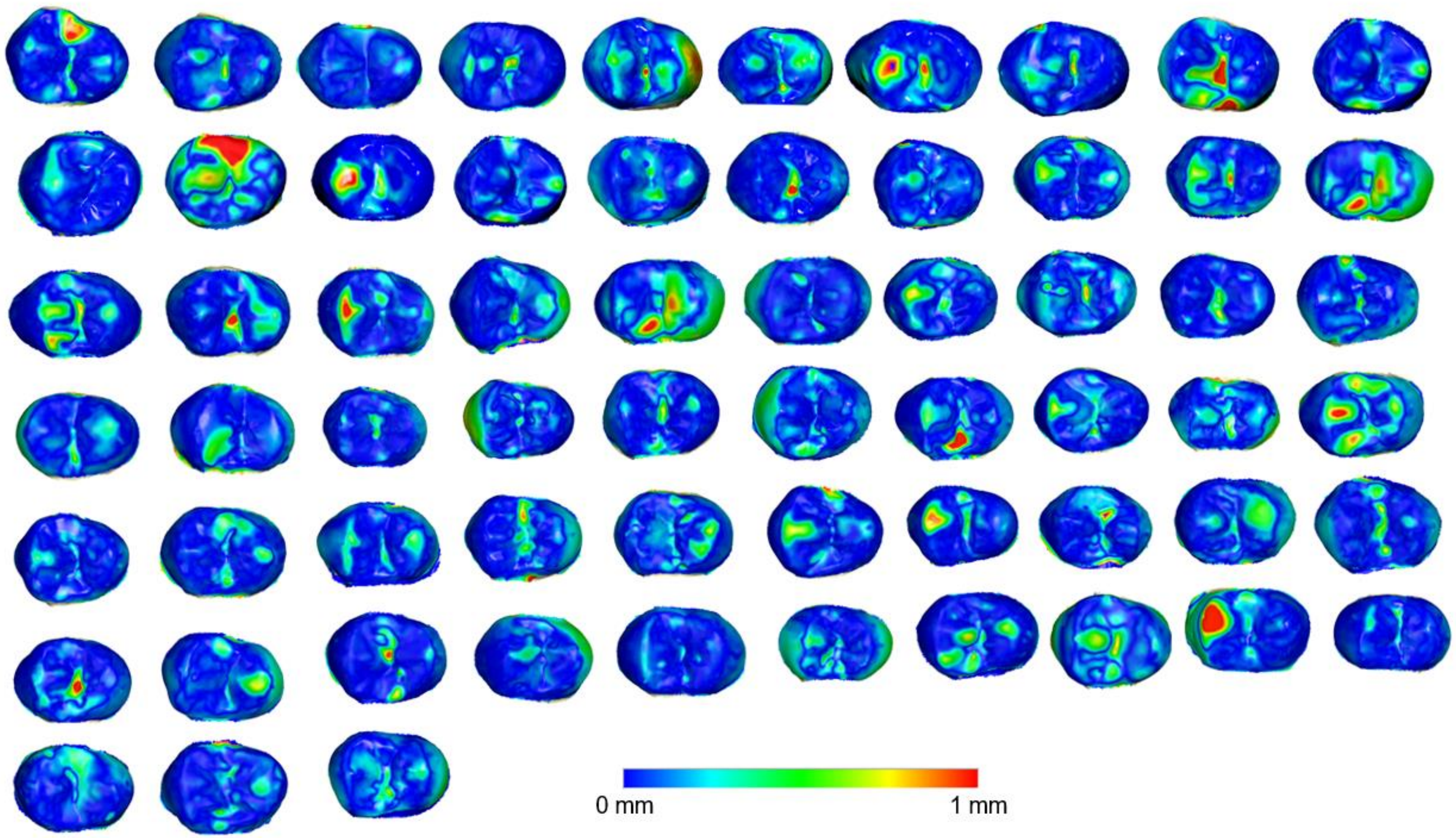

Supplementary Figure S6. Colour-coded distance maps illustrating all tooth wear patterns observed at the right maxillary second premolars.

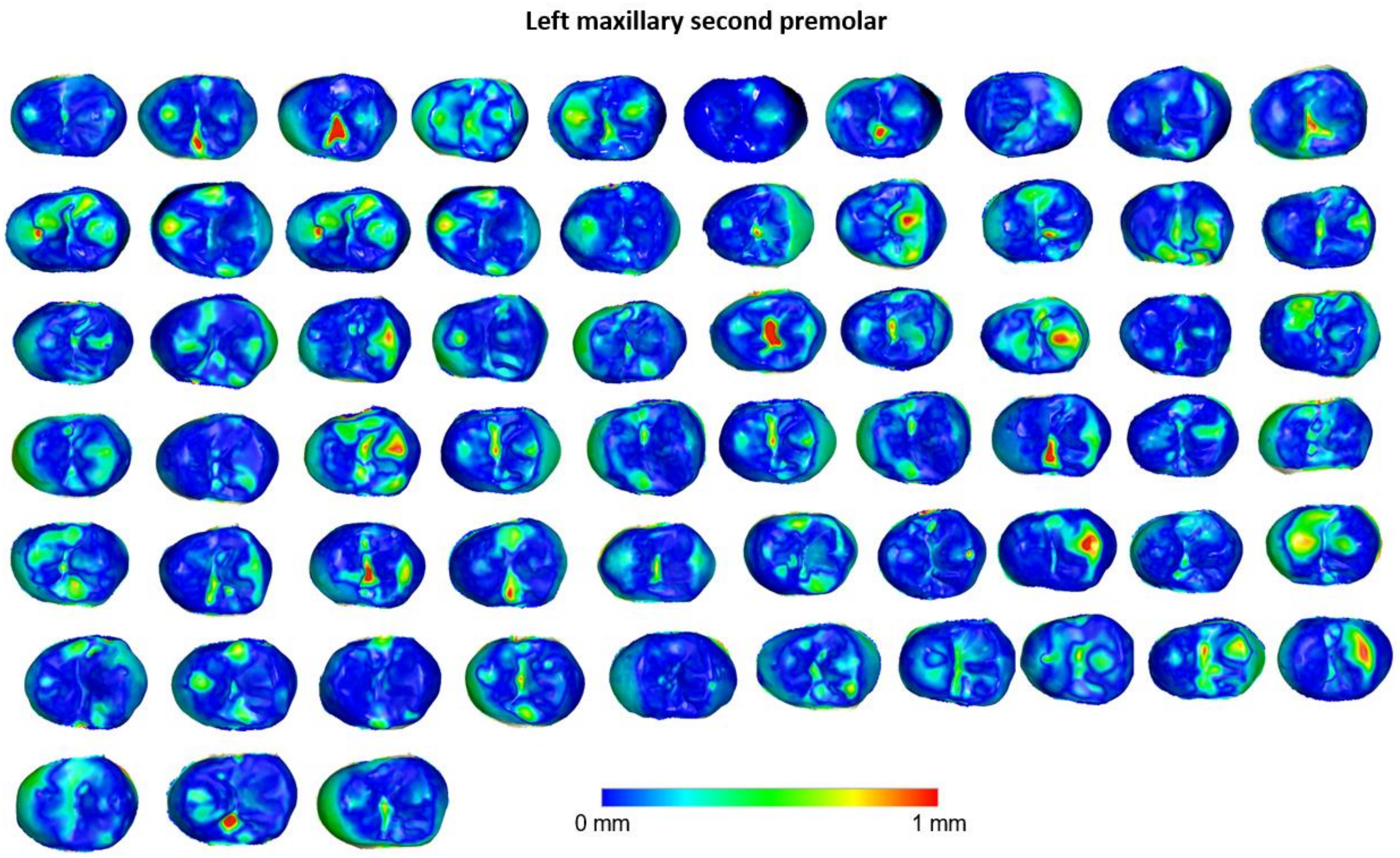

Supplementary Figure S7. Colour-coded distance maps illustrating all tooth wear patterns observed at the left maxillary second premolars.

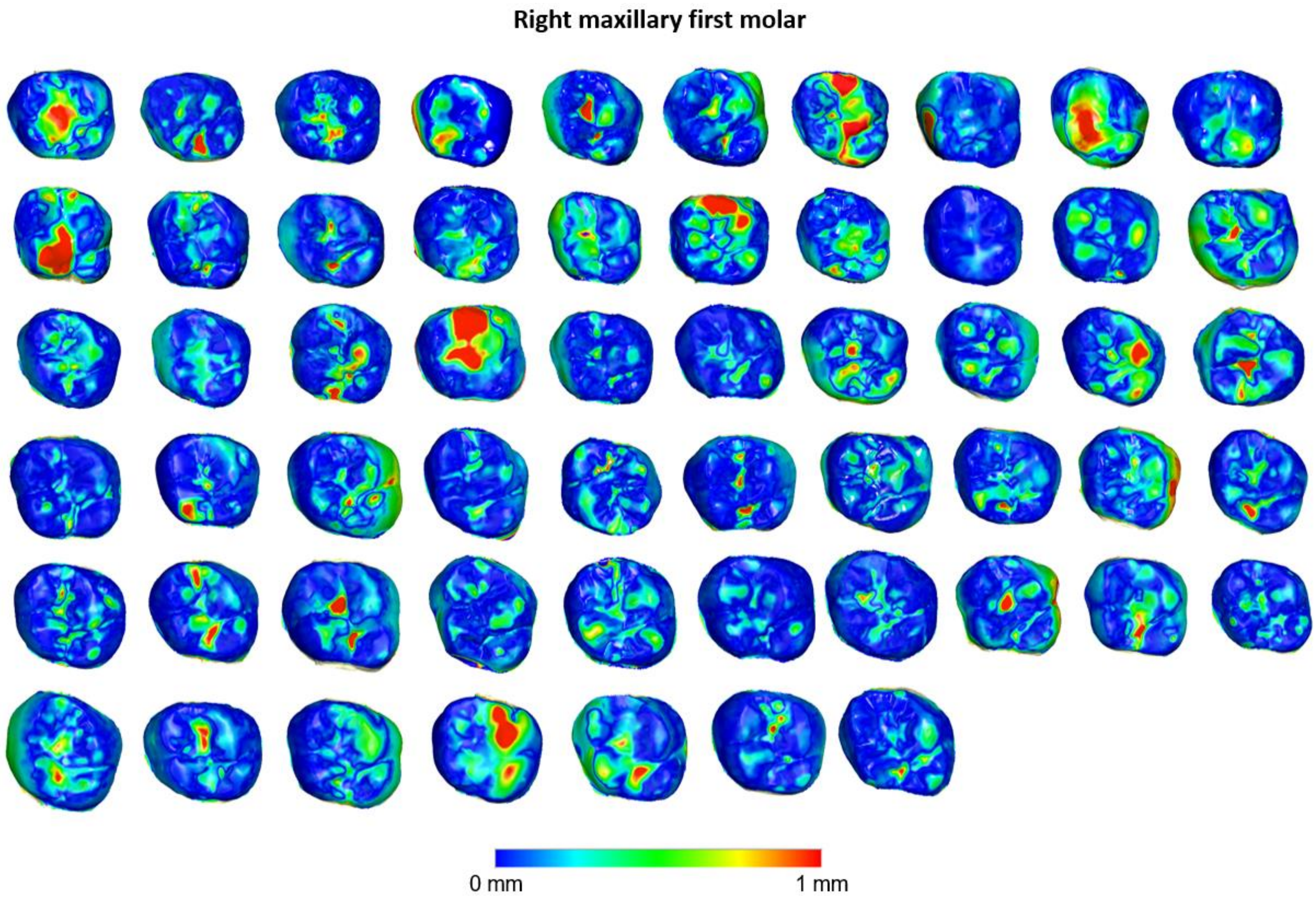

Supplementary Figure S8. Colour-coded distance maps illustrating all tooth wear patterns observed at the right maxillary first molars.

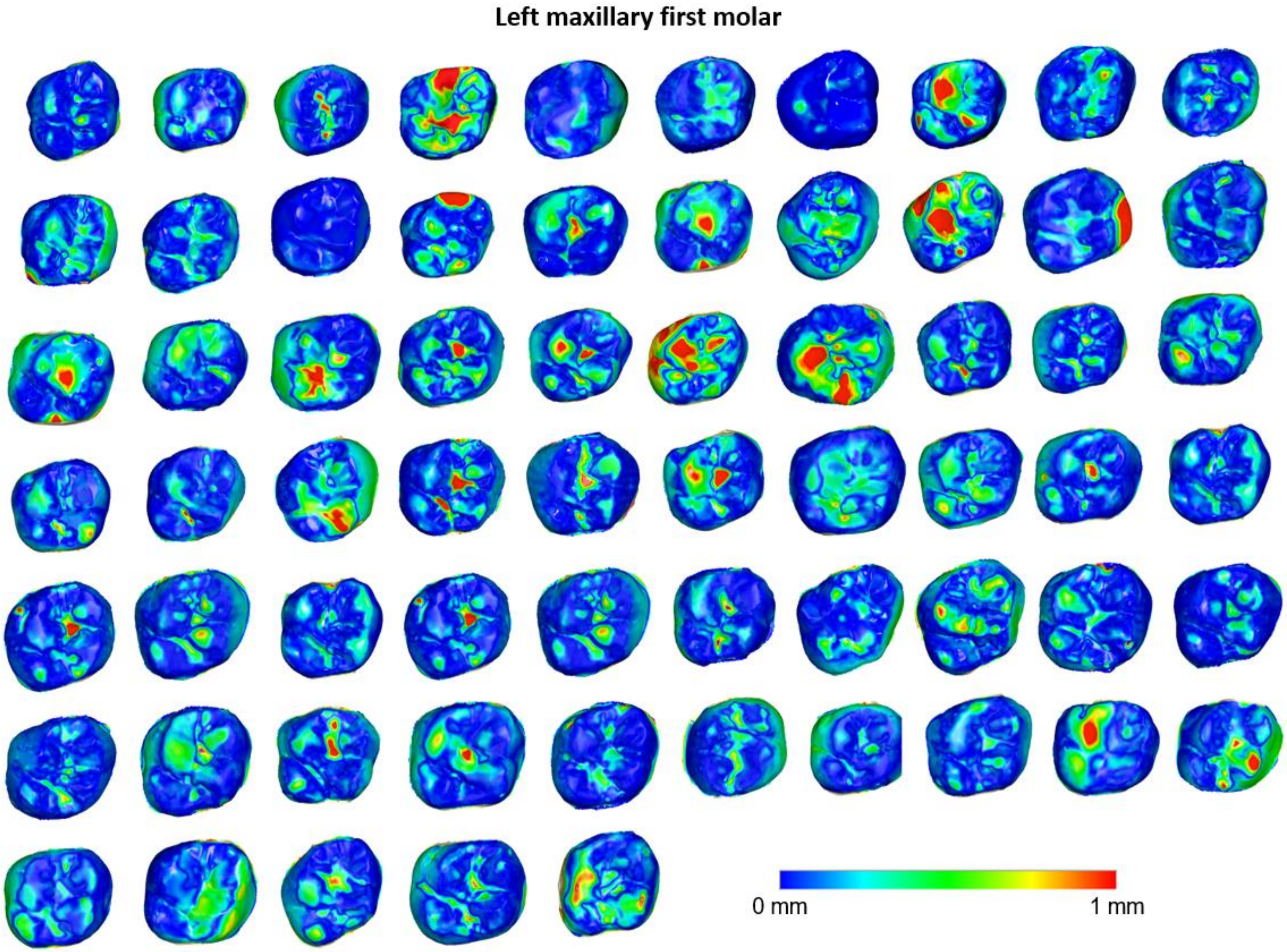

Supplementary Figure S9. Colour-coded distance maps illustrating all tooth wear patterns observed at the left maxillary first molars.

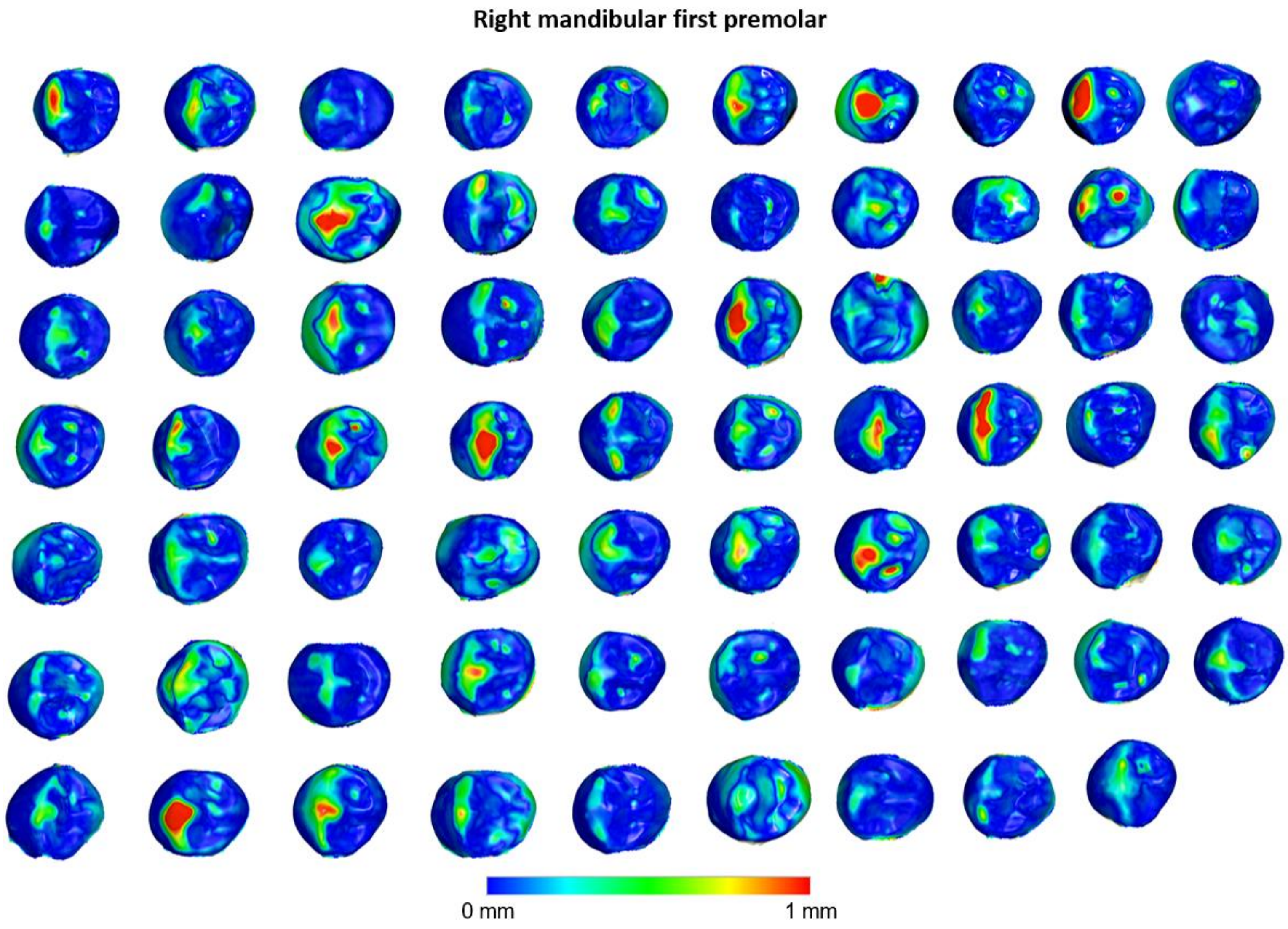

Supplementary Figure S10. Colour-coded distance maps illustrating all tooth wear patterns observed at the right mandibular first premolars.

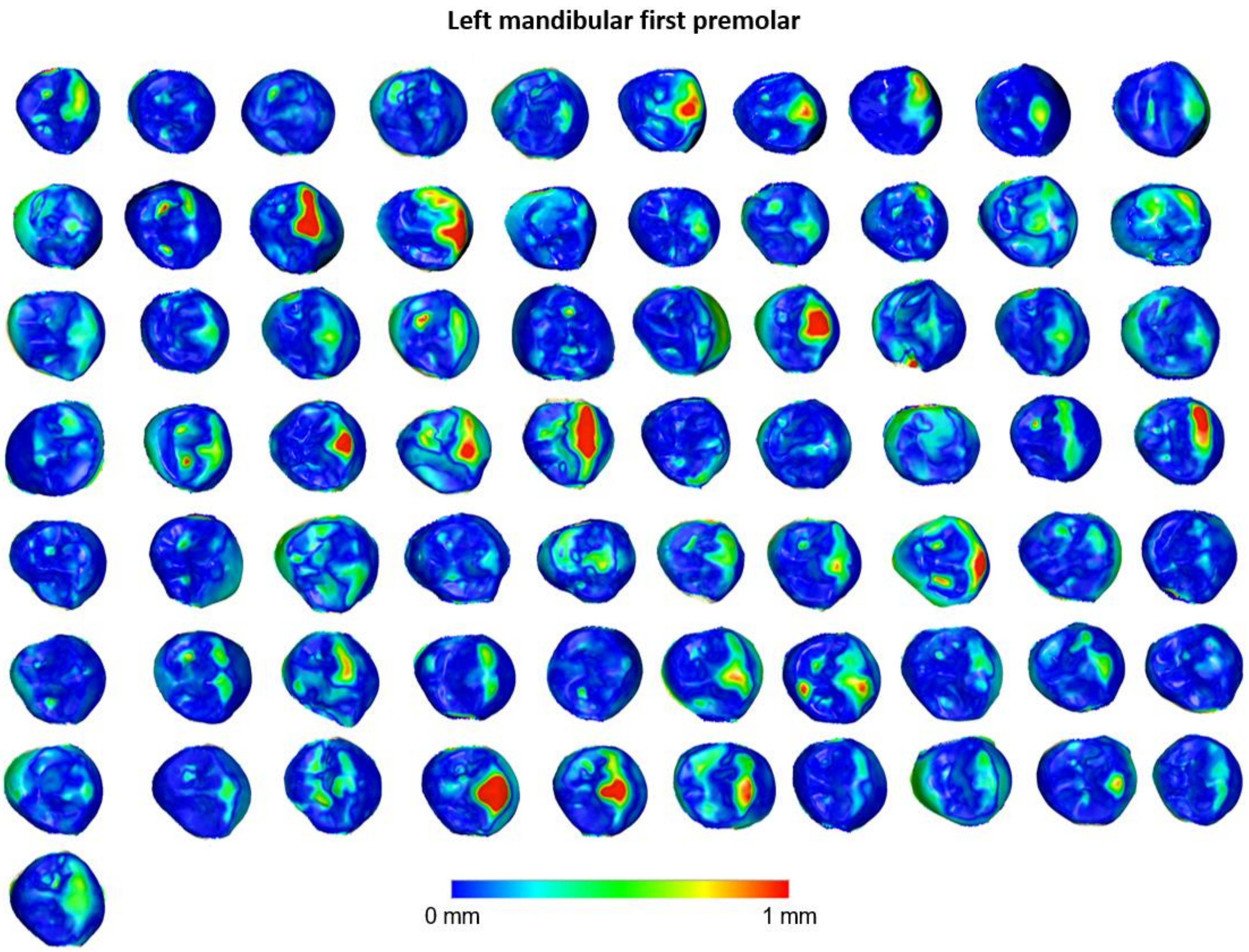

Supplementary Figure S11. Colour-coded distance maps illustrating all tooth wear patterns observed at the left mandibular first premolars.

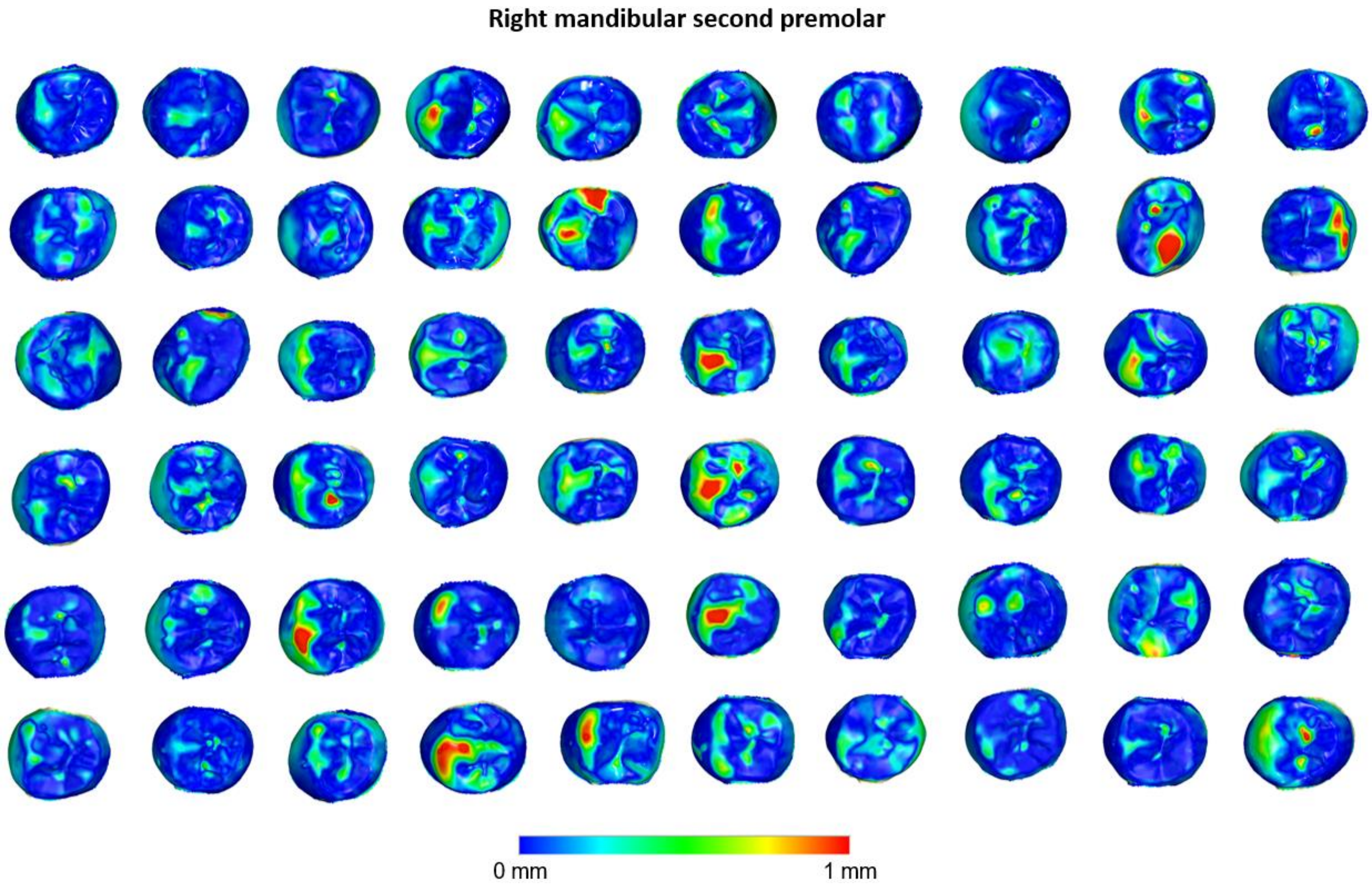

Supplementary Figure S12. Colour-coded distance maps illustrating all tooth wear patterns observed at the right mandibular second premolars.

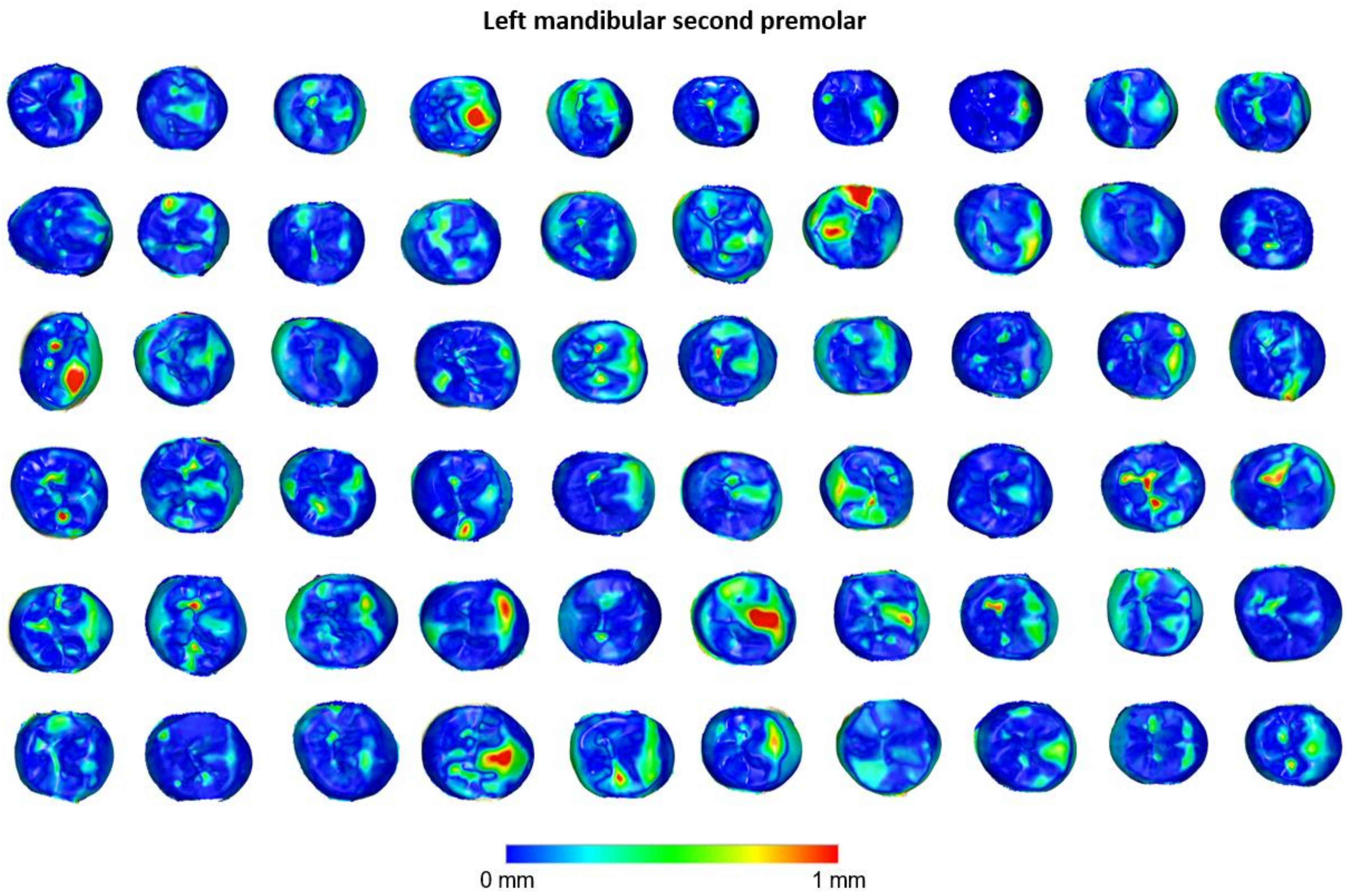

Supplementary Figure S13. Colour-coded distance maps illustrating all tooth wear patterns observed at the left mandibular second premolars.

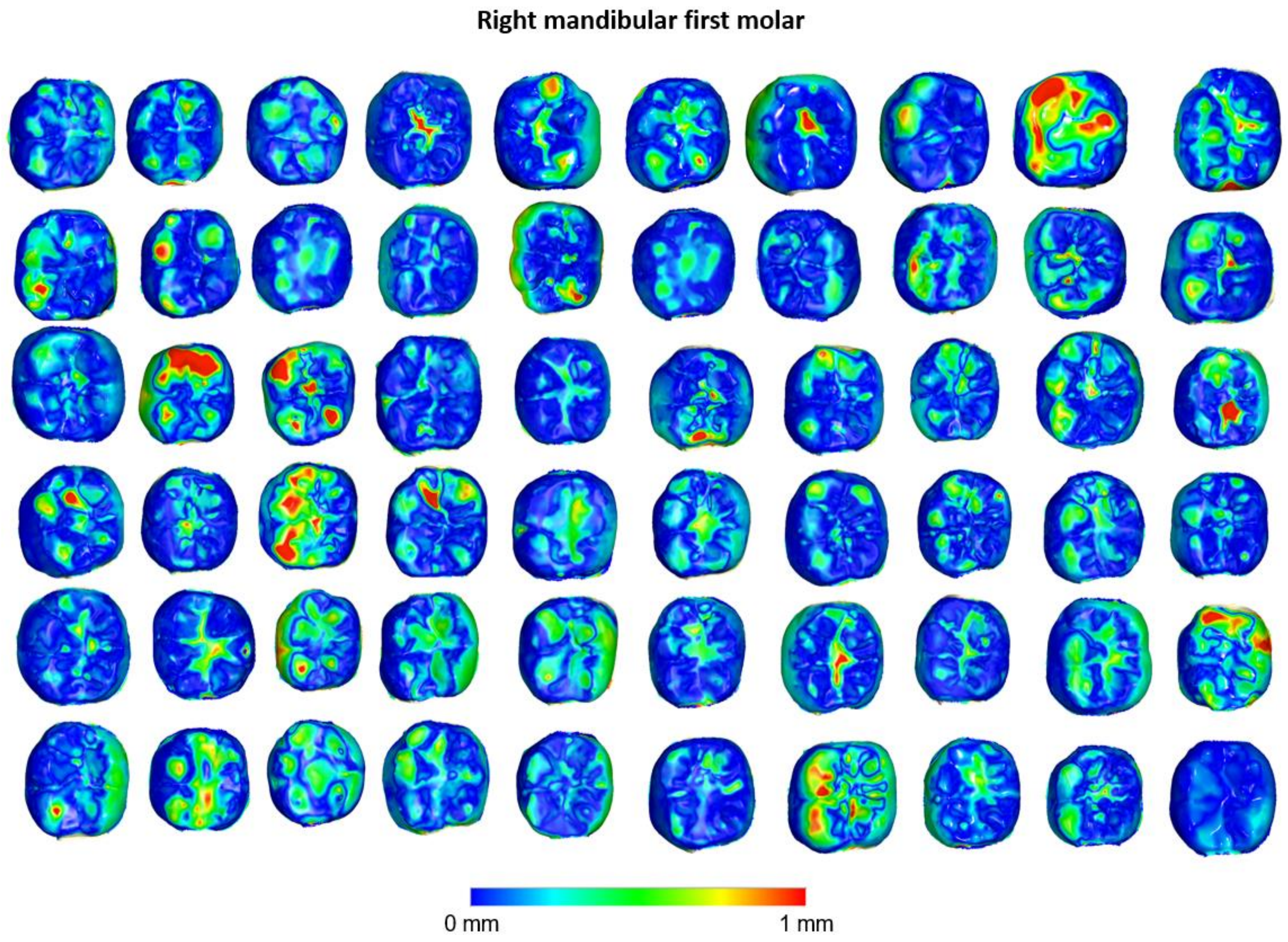

Supplementary Figure S14. Colour-coded distance maps illustrating all tooth wear patterns observed at the right mandibular first molars.

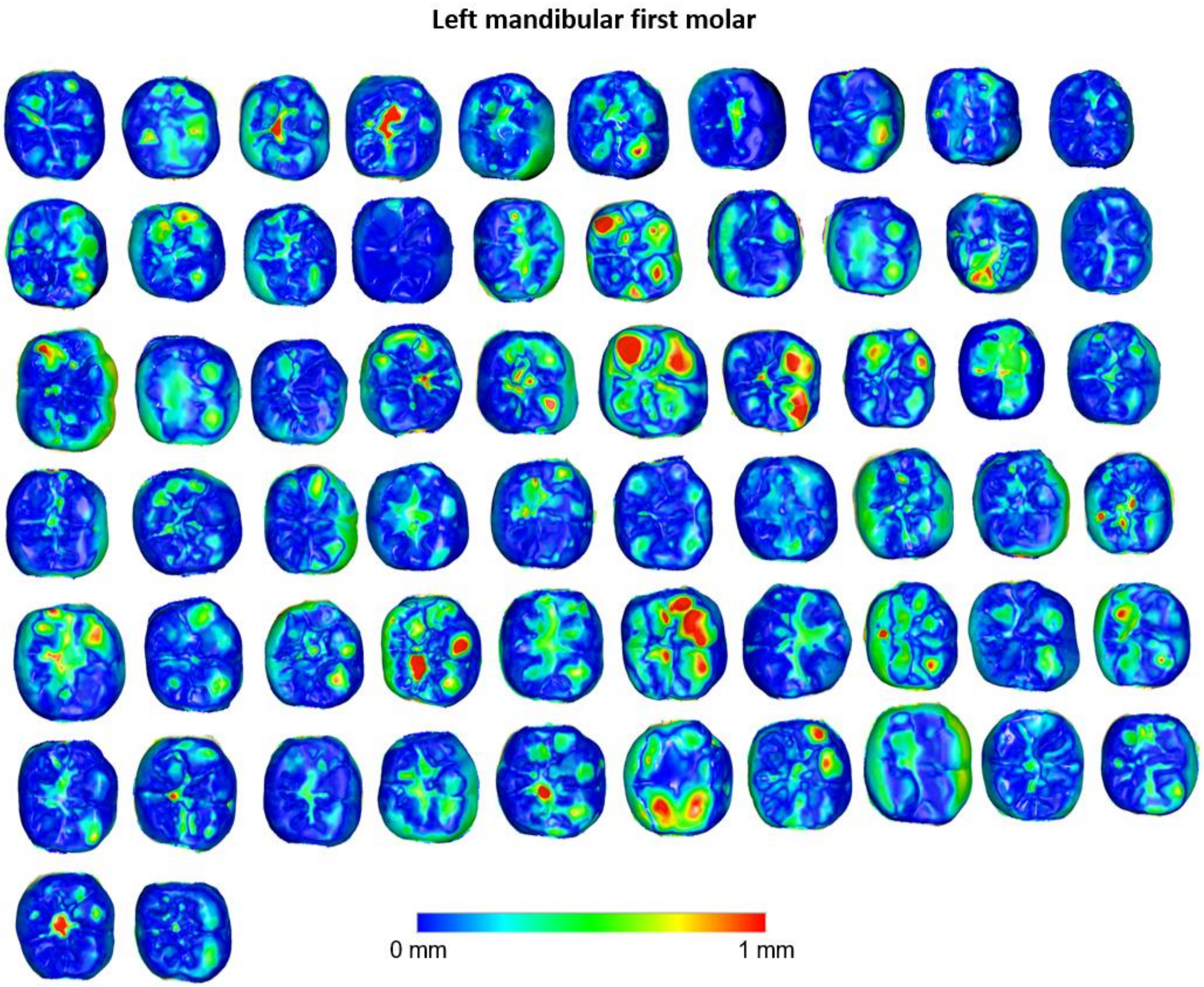

Supplementary Figure S15. Colour-coded distance maps illustrating all tooth wear patterns observed at the left mandibular first molars.
